# Dynamic changes in arterial blood gas during cardiopulmonary resuscitation in out-of-hospital cardiac arrest

**DOI:** 10.1038/s41598-021-02764-4

**Published:** 2021-11-30

**Authors:** Seok-In Hong, June-Sung Kim, Youn-Jung Kim, Won Young Kim

**Affiliations:** Department of Emergency Medicine, Asan Medical Center, University of Ulsan College of Medicine, Seoul, 05505 Korea

**Keywords:** Cardiovascular diseases, Prognosis

## Abstract

We aimed to investigate the prognostic value of dynamic changes in arterial blood gas analysis (ABGA) measured after the start of cardiopulmonary resuscitation (CPR) for return of spontaneous circulation (ROSC) in patients with out-of-hospital cardiac arrest (OHCA). This prospective observational study was conducted at the emergency department of a university hospital from February 2018 to February 2020. All blood samples for gas analysis were collected from a radial or femoral arterial line, which was inserted during CPR. Changes in ABGA parameters were expressed as delta (Δ), defined as the values of the second ABGA minus the values of the initial ABGA. The primary outcome was sustained ROSC. Out of the 80 patients included in the analysis, 13 achieved sustained ROSC after in-hospital resuscitation. Multivariable logistic analysis revealed that ΔpaO_2_ (odds ratio [OR] = 1.023; 95% confidence interval [CI] = 1.004–1.043, *p* = 0.020) along with prehospital shockable rhythm (OR = 84.680; 95% CI = 2.561–2799.939, *p* = 0.013) and total resuscitation duration (OR = 0.881; 95% CI = 0.805–0.964, *p* = 0.006) were significant predictors for sustained ROSC. Our study suggests a possible association between ΔpaO_2_ in ABGA during CPR and an increased rate of sustained ROSC in the late phase of OHCA.

## Introduction

Cardiac arrest results in various complications in the human body, including changes in acid–base status and blood gas, such as metabolic acidosis, hypoxia, and hypercarbia^1^. Arterial blood gas analysis (ABGA), as one of the intra-cardiopulmonary resuscitation (CPR) parameters, may provide useful diagnostic and prognostic clues in patients with out-of-hospital cardiac arrest (OHCA)^2^. The values identified on ABGA during CPR, such as arterial pH, lactic acid level, partial pressure of oxygen (paO_2_), and partial pressure of carbon dioxide (paCO_2_) have been thought to reflect tissue hypoxia and duration of hypoperfusion^3,4^.

The prognostication in patients with cardiac arrest for their immediate outcome, namely, return of spontaneous circulation (ROSC) and survival, requires consideration of numerous factors^5^. In addition to the pre-arrest factors such as witnessed cardiac arrest, bystander CPR, and the presence of shockable rhythms, parameters obtained during CPR might add more accuracy to the prognostication. Some studies have reported that pH and base excess (BE) are associated with ROSC in OHCA^6–8^. Recently, two studies have shown that higher levels of paO_2_ are significantly associated with increased rates of survival to hospital admission in OHCA^9,10^. Another study also reported that pCO_2_ level during CPR was an independent predictor for sustained ROSC in OHCA^11^. These results should be interpreted with caution owing to discordance among studies. Moreover, studies on the blood gas analysis during CPR have struggled with technical difficulties in obtaining blood samples, inconsistency in acquisition timing, and ambiguity as to whether the blood sample site was a vein or an artery^8^.

To date, a singular measurement of ABGA during CPR has not been able to provide accurate prognostication for patients with OHCA and previous investigations have not performed serial measurement of ABGA. We hypothesized that the dynamic changes in parameters of ABGA during CPR would be associated with sustained ROSC in patients with OHCA; thus, we investigated the prognostic value of serial ABGA of blood samples obtained from an arterial line.

## Methods

### Study design and setting

This prospective observational study was conducted at the emergency department (ED) of a university hospital, which, at the time of writing of this paper, had an annual census of approximately 120,000 visits. The present study was based on the Korean Cardiac Arrest Research Consortium (KoCARC) registry between February 2018 and February 2020. The KoCARC is a multicenter collaborative research network in Korea^12^. The KoCARC registry was registered at clinicaltrials.gov as protocol NCT03222999. All patients with OHCA other than traumatic OHCA were enrolled in the KoCARC registry in sequence. The participating institutions had to record the values of serial ABGAs; however, placement of the arterial line during CPR was not mandatory. Therefore, we used the dataset of a single institution (Asan Medical Center) to guarantee methodological quality in medical practices (i.e., arterial line insertion and a record of the time when the blood samples were obtained from patients).

### Study population and data collection

In Korea, after one cycle of CPR, emergency medical service (EMS) providers are encouraged to “scoop and run” to the ED as soon as possible while they continue CPR during ambulance transport. EMS providers are not legally allowed to declare death in the field unless there are obvious signs of death (i.e., decapitation, decomposition, postmortem lividity, postmortem rigidity, and burnt beyond recognition).

The study population consisted of all consecutive nontraumatic adult (age ≥ 18 years) OHCA patients in the KoCARC registry who visited the ED. Contrary to the medical cause of cardiac arrest, traumatic cardiac arrest has different pathophysiology and treatment priorities^13^. The outcome of traumatic cardiac arrest has been associated with different factors, such as the severity of injury and the presence of reversible etiologies^14^. Thus, patients with traumatic cardiac arrest were excluded from this study. Other exclusion criteria included if a blood sample was not obtained at all or not obtained from the arterial line, or any ROSC was achieved before second ABGA, or second ABGA data were missing. Achievement of ROSC was declared when the patient had a palpable pulse in the absence of chest compressions regardless of the duration and sustained ROSC was declared when the patient had a palpable pulse for > 20 min. Data for analysis were obtained from EMS reports and medical records. Clinically important baseline characteristics were chosen based on prior literature^11,15,16^. We extracted the data for demographic and arrest characteristics, initial and second ABGA at ED, and outcome (achievement of sustained ROSC) for the patients enrolled in the study^17^.

### In-hospital management and blood sampling

Basic and advanced cardiovascular life support were performed in accordance with the Advanced Cardiac Life Support guidelines of 2015^18^. EMS personnel provided basic and advanced cardiovascular life support as per the guidelines in the prehospital stage of resuscitation. After arrival at the ED, all patients were intubated and ventilated with an Ambu bag using 100% oxygen at a flow rate of 15 L/min, and they were provided high-quality CPR recommended by the guidelines as much as possible (i.e., minimizing interruptions in chest compressions, providing compressions of adequate rate and depth, avoiding leaning on the chest between compressions, and avoiding excessive ventilation). To maintain the quality of CPR, we used a metronome, which helped maintain the rate of manual chest compression and ventilation. A mechanical chest compression device (LUCAS^®^, Lund University Cardiopulmonary Assist System; Physio-Control Inc./Jolife AB, Lund, Sweden or AutoPulse^®^; Zoll Medical Corporation, Chelmsford, MA, USA) was used occasionally either in the pre- or the in-hospital stage of resuscitation. Further, a CPR leader observed all the situations and checked the quality of ongoing CPR. However, objective CPR feedback devices were not used in these cases.

Patients were required to undergo arterial catheterization and arterial blood pressure monitoring: this has been mandated for all OHCA patients in our institution since 2018. Ultrasonography-guided arterial catheterization was performed by an emergency physician who was on duty when the patient arrived at the ED. All the physicians involved were doctors who had worked at our institution for more than 3 years and who were skilled enough for ultrasonography-guided catheterization. Arterial catheterization was performed as soon as possible, and the placement was confirmed by the presentation of the arterial wave during CPR. Arterial waves representing the pulse pressure generated by chest compressions were discriminated from venous waves that were affected by respiratory variation and which showed lower pressure^19^.

Blood samples for gas analysis were collected from the radial or femoral arterial line using sodium-heparin-coated syringes. The first arterial blood sample was obtained from the arterial line. The second consecutive ABGA was conducted 10 min after the start of CPR at the ED, not from the time when the initial blood sample was obtained from the patient. It was considered appropriate to collect a second blood sample at 10 min since more frequent blood sampling was not suitable in real practice. Involved physicians were not blinded to the blood gas analysis and they were able to choose appropriate treatment options according to the results of blood gas analysis. The physicians could terminate resuscitation efforts only if the guardian wanted to, otherwise, they continued the resuscitation efforts for at least 20 min after the ED arrival of the patient. Changes in ABGA values were expressed as delta (Δ) and these were defined as the values of the second ABGA minus the values of the first ABGA. ABGA was performed using RAPIDPoint 500 (Siemens Healthineers, Erlangen, Germany). The reporting ranges of each value were as follows: pH, 6.50–7.80; paO_2_, 0.0–760.0 mmHg; paCO_2_, 5.0–115.0 mmHg; lactic acid level, 0.3–15.0 mmol/L; bicarbonate (HCO_3_) level, 3.0–60.0 mmol/L; and BE, − 99.9 to 99.9 mmol/L. Test results were available within 60 s. In cases where the values exceeded their instrument limit, those values were assumed as the limit values.

### Statistical analysis

Data were presented as numbers with percentages for categorical variables and median with interquartile range (IQR) for continuous variables. Variables were tested for normality of distribution using a Kolmogorov–Smirnov test. The values of non-normally distributed continuous variables were compared by a Mann–Whitney U test. Differences between categorical variables were analyzed by a chi-square test or Fisher’s exact test, as appropriate. Clinically significant baseline characteristics and baseline ABGA values as potential predictors of ROSC were first examined using univariable logistic analysis. Multivariable logistic analysis was performed using the stepwise backward elimination method for the same variables examined in the univariable logistic analysis. The results of the logistic regression analysis were summarized by estimating the odds ratios (ORs) and the respective 95% confidence intervals (CIs). A Hosmer–Lemeshow test was employed for logistic regression models. A two-tailed *p* value of < 0.05 was considered statistically significant and adjustments for multiple comparisons were also performed. All statistical analyses were performed using SPSS Statistics for Windows, version 21.0 (IBM Corp., Armonk, NY, USA).

### Ethical approval and informed consent

The KoCARC data collection protocol of this study was approved by the Institutional Review Board and the Ethics Committee of Asan Medical Center (Approval number: 2015-1224). The requirement for informed consent was waived in the present study under the following circumstances: the study involved no more than minimal risk to subjects; the study could not practicably be carried out without the waiver; the waiver or alteration would not adversely affect the rights and welfare of the subjects; whenever appropriate, the subject or legal guardian was provided with additional pertinent information after participation. All methods were performed in accordance with the relevant guidelines and regulations.

## Results

**Figure 1:**
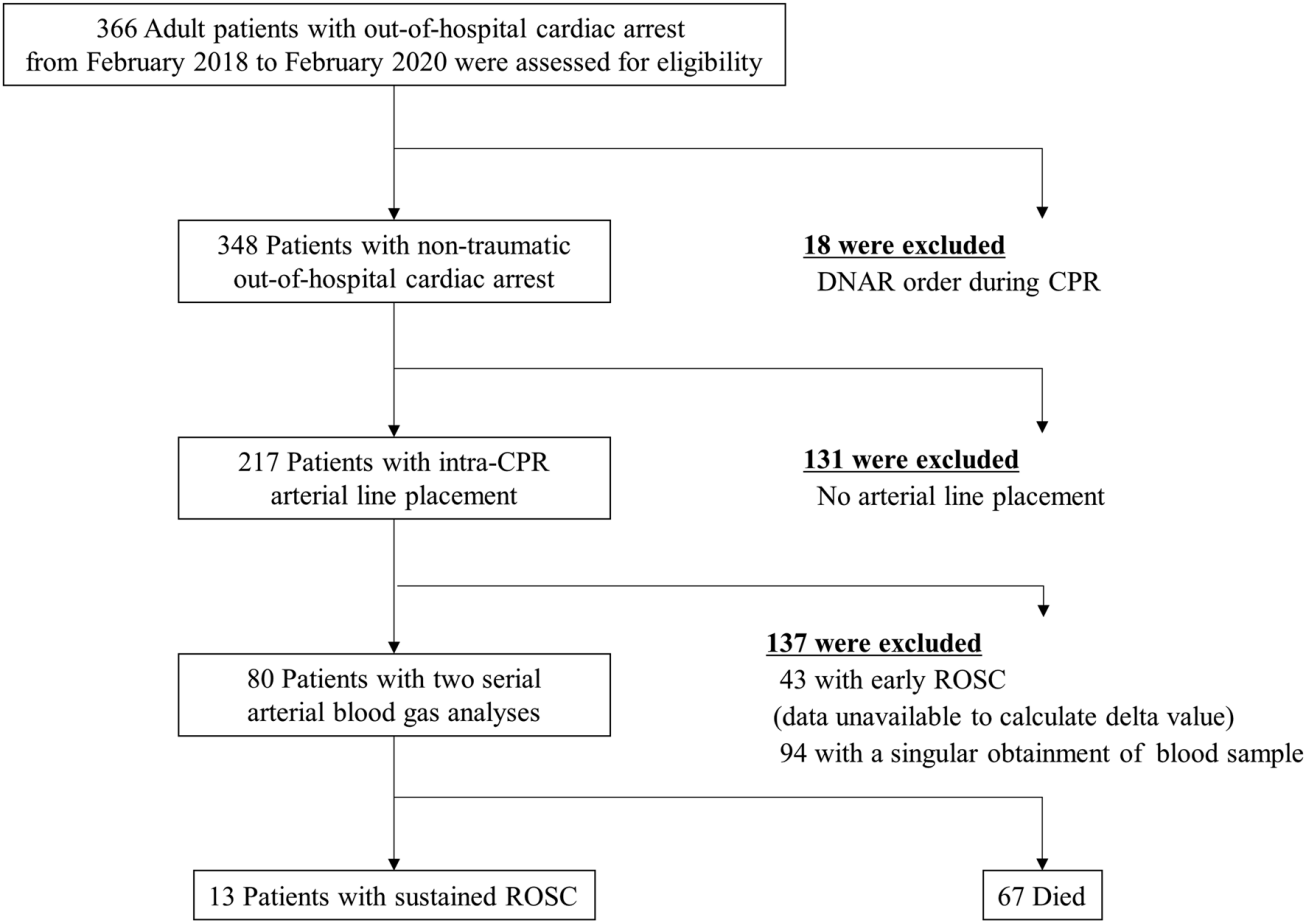
Patient flow chart. To calculate the delta values of ABGA parameters, two serial ABGAs were obtained for each patient included in the study. *ABGA* arterial blood gas analysis, *CPR* cardiopulmonary resuscitation, *DNAR* do not attempt resuscitation, *ROSC* return of spontaneous circulation.

During the study period, 366 adult OHCA patients arrived at our ED; of these patients, 348 with non-traumatic cardiac arrest who were resuscitated in the ED were assessed for eligibility. An arterial line was not placed in 131 patients during CPR; thus, they were excluded from the study. An additional 137 patients whose second ABGA data were unavailable due to missing values or early ROSC were also excluded from the study. Finally, the dataset for 80 patients was analyzed, and 13 patients achieved sustained ROSC after in-hospital resuscitation (Fig. 1).

A comparison of baseline and arrest characteristics of the included patients is shown in Table 1. The median age of the patients who achieved sustained ROSC was higher than that of the patients who did not survive, although there was no significant difference. Male predominance and total resuscitation duration were significantly different between both groups. Five out of 13 patients (39%) were male in the sustained ROSC group, compared to 48 out of 67 patients (72%) in the no-ROSC group (*p* = 0.028). Although there was no significant difference in prehospital downtime between the two groups, total resuscitation duration was shorter in the sustained ROSC group, with a median duration of 41 (IQR, 34–48) min versus 53 (44–66) min in the no-ROSC group (*p* = 0.002). Other arrest characteristics were not significantly different, including the level of end-tidal carbon dioxide in both groups.

**Table 1:** Baseline characteristics of included patients and arterial blood gas parameters obtained during cardio-pulmonary resuscitation (CPR).

Characteristics^a^	Sustained ROSC (n = 13)	No ROSC (n = 67)	*p* value
**Arrest characteristics**			
Age (years)	75 (62–80)	71 (62–80)	0.779
Male sex	5 (39)	48 (72)	0.028
Witnessed	11 (85)	51 (76)	0.722
Bystander CPR	9 (69)	45 (67)	1.000
Prehospital use of mechanical compression device^b^	4 (31)	15 (22)	0.496
In-hospital use of mechanical compression device^c^	8 (62)	35 (52)	0.762
Prehospital shockable rhythm	3 (23)	13 (19)	0.717
Presumed cardiac cause	5 (39)	23 (38)	1.000
Prehospital downtime (mins)^d^	26 (24–33)	29 (24–37)	0.321
Total resuscitation duration (mins)	41 (34–48)	53 (44–66)	0.002
ETCO_2_ (mmHg)^e^	10 (8–17)	13 (9–20)	0.320
**Initial arterial blood gas parameters**			
Time to analysis (mins)	4 (3–4)	3 (2–4)	0.759
pH	6.91 (6.80–6.98)	6.83 (6.69–6.97)	0.194
paO_2_ (mmHg)	25.7 (15.2–49.4)	47.5 (25.5–69.2)	0.099
paCO_2_ (mmHg)	59.4 (47.7–79.8)	69.8 (53.4–83.5)	0.365
Lactic acid (mmol/L)	9.6 (8.0–14.8)	12.9 (9.9–15.0)	0.193
HCO_3_ (mmol/L)	10.9 (7.7–17.7)	10.2 (7.4–13.9)	0.451
BE (mmol/L)	− 20.7 (− 24.5 to − 14.7)	− 22.0 (− 27.3 to − 18.6)	0.113
**Second arterial blood gas parameters**			
Time to analysis (mins)	12 (11–14)	12 (11–15)	0.927
pH	6.87 (6.71–6.94)	6.87 (6.72–7.01)	0.523
paO_2_ (mmHg)	103.1 (94.3–144.3)	68.2 (43.8–100.8)	0.001
paCO_2_ (mmHg)	56.5 (48.7–61.0)	71.1 (50.8–95.1)	0.159
Lactic acid (mmol/L)	9.8 (9.1–12.6)	12.9 (10.5–15.0)	0.171
HCO_3_ (mmol/L)	8.8 (5.0–11.0)	11.1 (7.3–15.9)	0.089
BE (mmol/L)	− 22.5 (− 28.1 to − 21.8)	− 21.5 (− 26.5 to − 14.0)	0.235

*BE* base excess, *ETCO*_*2*_ end-tidal carbon dioxide, *HCO*_*3*_ bicarbonate, *paO*_*2*_ partial pressure of arterial oxygen, *paCO*_*2*_ partial pressure of arterial carbon dioxide, *ROSC* return of spontaneous circulation.

^a^Continuous variables are expressed as median with interquartile ranges; categorical values are expressed as a number with a percentage.

^b^LUCAS^®^, Lund University Cardiopulmonary Assist System; Physio-Control Inc./Jolife AB, Lund, Sweden or AutoPulse^®^; Zoll Medical Corporation, Chelmsford, MA, USA.

^c^LUCAS^®^.

^d^Defined as the estimated time from the first recognition of a sign of cardiac arrest to arrive in the hospital. The downtime in unwitnessed cases would be longer than the estimation.

^e^The level of ETCO_2_ was measured at 10 min after ED arrival of the patient.

### ABGAs and their dynamic changes during CPR

Initial and second ABGAs values are presented in Table 1. There was no significant difference between both groups in time to analysis for ABGA. None of the initial or second parameters reached statistical significance, except for median paO_2_ in the second ABGA; median paO_2_ in the second ABGA was 103.1 (94.3–144.3) mmHg in the sustained ROSC group, which was significantly higher than that of the no-ROSC group (68.2 [43.8–100.8] mmHg; *p* = 0.001).

**Figure 2:**
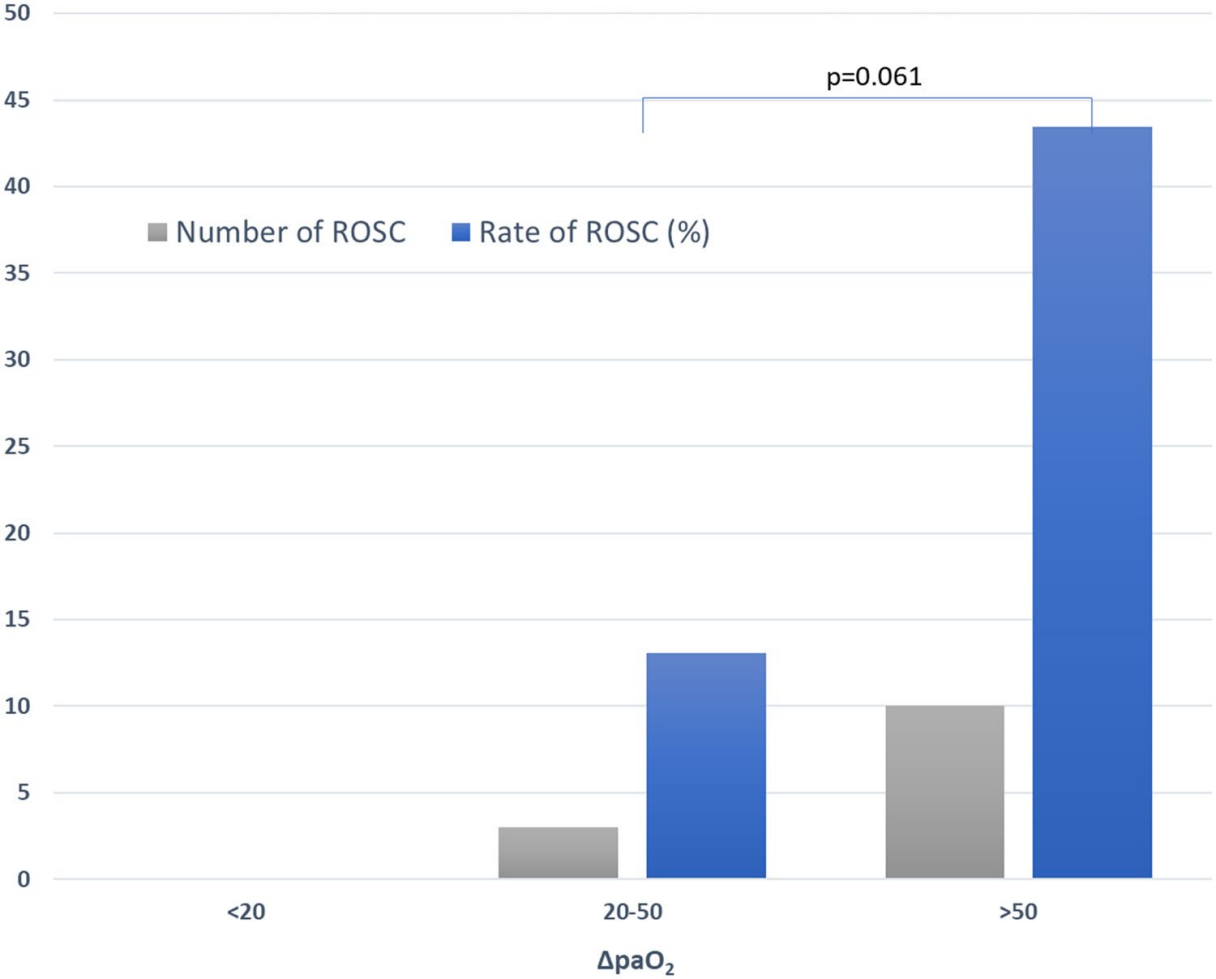
Rates of sustained return of spontaneous circulation (ROSC) with respect to the delta values of arterial pO_2_ level. The delta values were divided into three groups. Cutoff values for each group were chosen arbitrarily. Note that there was no sustained ROSC case in the first group (ΔpaO_2_ < 20 mmHg). *pO*_*2*_ partial pressure of oxygen.

The delta values between both serial ABGAs were calculated by subtracting the parameters of the initial ABGA from the second ABGA. Median time differences between the serial ABGAs were approximately 9 min and were not significantly different between the groups. The ΔpH, ΔpaO_2_, ΔHCO_3_, and ΔBE values showed significant differences between the groups. The patients who achieved sustained ROSC showed a profound increase in median paO_2_ compared to that in patients who did not achieve sustained ROSC (90.2 [51.2–122.7] vs. 16.5 [− 0.4 to 42.2] mmHg, respectively; *p* < 0.001). There were greater decreases in pH and HCO_3_ in the sustained ROSC group (− 0.09 [− 0.13 to − 0.04] and − 2.2 [− 6.7 to − 0.7] mmol/L, respectively) than in the no-ROSC group (− 0.01 [− 0.08 to 0.09] and − 0.7 [− 2.8 to 5.2] mmol/L; *p* = 0.038 and 0.019, respectively). Median ΔBE was − 3.6 (− 7.4 to − 2.5) mmol/L in sustained ROSC patients, which was slightly higher when compared to that in the no-ROSC patients (− 4.0 [− 4.1 to 5.8] mmol/L; *p* = 0.007) (Table 2). Notably, division of the patients based on an arbitrary value of ΔpaO_2_ revealed that patients with ΔpaO_2_ < 20 mmHg did not achieve ROSC (Fig. 2).

**Table 2:** Delta values of parameters between the initial and second arterial blood gas analysis.

Delta values of arterial blood gas parameters^†^	Sustained ROSC (n = 13)	No ROSC (n = 67)	*p* value
Analysis time difference (mins)	9 (8–10)	9 (7–11)	0.798
ΔpH	− 0.09 (− 0.13 to − 0.04)	− 0.01 (− 0.08 to 0.09)	0.038
ΔpaO_2_ (mmHg)	90.2 (51.2–122.7)	16.5 (− 0.4 to 42.2)	< 0.001
ΔpaCO_2_ (mmHg)	0.0 (− 22.7 to 10.6)	3.4 (− 16.8 to 21.3)	0.485
ΔLactic acid (mmol/L)	1.1 (− 0.8 to 2.9)	0.1 (− 0.7 to 1.4)	0.497
ΔHCO_3_ (mmol/L)	− 2.2 (− 6.7 to − 0.7)	− 0.7 (− 2.8 to 5.2)	0.019
ΔBE (mmol/L)	− 3.6 (− 7.4 to − 2.5)	− 4.0 (− 4.1 to 5.8)	0.007

*BE* base excess, *HCO*_*3*_ bicarbonate, *paO*_*2*_ partial pressure of arterial oxygen, *paCO*_*2*_ partial pressure of arterial carbon dioxide, *ROSC* return of spontaneous circulation.

^†^Variables are expressed as median with interquartile ranges.

Univariable logistic analyses for known arrest characteristics and the delta values of ABGA showed similar results as aforementioned (Table 3A). In multivariable logistic analysis, sex, prehospital shockable rhythm, total resuscitation duration, ΔpaO_2_, and ΔBE remained in the final model of sustained ROSC. In the final model, ΔpaO_2_ (OR, 1.023; CI, 1.004–1.043; *p* = 0.020), prehospital shockable rhythm (OR, 84.680; CI, 2.561–2799.939; *p* = 0.013), and total resuscitation duration (OR, 0.881; CI, 0.805–0.964; *p* = 0.006) were significant predictors for sustained ROSC (Table 3B).

**Table 3:** Prognostic factors of included patients and the delta values of arterial blood gas parameters.

	OR	95% CI	*p* value
**(A) Univariable analysis**			
Male sex	0.247	0.072–0.853	0.027
Age (years)	1.009	0.965–1.055	0.699
Witnessed	1.725	0.346–8.614	0.506
Bystander CPR	1.100	0.305–3.970	0.884
Prehospital shockable rhythm	1.246	0.300–5.182	0.762
Total resuscitation duration (mins)	0.933	0.885–0.985	0.011
Presumed cardiac cause	1.005	0.293–3.449	0.993
ETCO_2_ (mmHg)	0.963	0.879–1.054	0.413
ΔpH	0.013	0.000–1.652	0.079
ΔpaO_2_ (mmHg)	1.012	1.003–1.020	0.006
ΔpaCO_2_ (mmHg)	0.992	0.971–1.014	0.465
ΔHCO_3_ (mmol/L)	0.826	0.706–0.966	0.017
ΔBE (mmol/L)	0.828	0.707–0.970	0.019
**(B) Multivariable analysis**			
Male sex	0.217	0.024–1.994	0.177
Prehospital shockable rhythm	84.680	2.561–2799.939	0.013
Total resuscitation duration (mins)	0.881	0.805–0.964	0.006
ΔpaO_2_ (mmHg)	1.023	1.004–1.043	0.020
ΔBE (mmol/L)	0.871	0.711–1.068	0.185

The endpoint for all calculations was sustained return of spontaneous circulation (ROSC).

*BE* base excess, *CI* confidence interval, *CPR* cardiopulmonary resuscitation, *ETCO*_*2*_ end-tidal carbon dioxide, *HCO*_*3*_ bicarbonate, *OR* odds ratio, *paO*_*2*_ partial pressure of arterial oxygen, *paCO*_*2*_ partial pressure of arterial carbon dioxide.

(A) Univariable logistic analysis for the variables with assumed prognostic relevance. (B) Multivariable logistic analysis (stepwise backward elimination using likelihood ratio) for the variables presented in (A).

## Discussion

In the present study, high delta paO_2_ between the initial and second ABGAs along with prehospital shockable rhythm and short resuscitation duration during CPR was significantly associated with an increased rate of sustained ROSC in patients with OHCA. A singular ABGA measurement during CPR had been unable to provide accurate prognostication for outcomes in patients with OHCA. Although the relationships between ABGA parameters and OHCA outcomes have been investigated in several studies, the contribution of these studies was limited due to the uncertainty of the blood sample source^8,9,11^. On the contrary, all blood samples for analysis were obtained from arterial lines in this study. To the best of our knowledge, this is the first report detailing the dynamic changes in ABGA during ongoing CPR in humans.

Our study showed that delta paO_2_ was significantly associated with sustained ROSC. There are other previous studies that investigated the relationship between paO_2_ during CPR and the rate of survival to hospital admission^9,10^. Although these results came from analysis of singular ABGA during CPR, the findings suggest that high oxygen tension in the blood may reflect adequate ventilation and sufficient circulation. Alternatively, high paO_2_ during CPR may be a surrogate for the severity of the underlying medical illnesses. Although the physiologic mechanisms by which higher paO_2_ results in higher rates of ROSC is not clear, the clinical implications of our study results can be suggested as follows: As a means of determining continuous resuscitation, high ΔpaO_2_ during CPR may be considered as a selection criterion for patients requiring prolonged resuscitation efforts. On the other hand, there is one result to be interpreted with caution, namely, that patients with ΔpaO_2_ < 20 mmHg do not achieve ROSC. In this case, 20 mmHg was an arbitrary value, not a statistically calculated one. We intended to show that patients with ΔpaO_2_ below a specific cutoff did not survive; however, this cutoff would vary depending on the setting of population, the duration of resuscitation, ABGA time, etc. Therefore, a cutoff of 20 mmHg could only be acceptable as a reference, and it could not be used to determine termination of resuscitation.

The level of paO_2_ might change depending on the oxygen supplied and the circulation generated by chest compression. We assumed that prehospital resuscitation was also performed with good quality; however, there was room for it not to be done as such. That was because the median value of the initial paO_2_ level was lower in the sustained ROSC group than in the other group (25.7 vs. 47.5 mmHg, *p* = 0.099) although it did not reach statistical significance. Otherwise, by minimizing confounders and following the guideline, all patients in the current study were intubated and ventilated with 100% oxygen, and were provided high-quality CPR as was possible after arrival at the ED. It seems appropriate to maintain 100% oxygen supply during CPR, despite the fact that high fraction of inspired oxygen (FiO_2_) may increase oxidative stress and release of reactive oxygen species, which result in molecular damages and a higher risk of mortality, especially post-ROSC^1,20–22^. Results are conflicting in terms of specific oxygen regimens that can increase the chance of survival. Further research is warranted to determine the optimal level of FiO_2_ during CPR.

Interestingly, arterial pH, HCO_3_ level, and BE had a greater decrease in the sustained ROSC group during CPR than in the no-ROSC group, although they were not found to be statistically significant in the multivariable logistic analysis. A metabolic component was present in most of the cases; however, hypercarbia still largely contributed to acidosis. All study patients were intubated and ventilated manually according to the 2015 guidelines regardless of the parameters of the first ABGA^18^, however, the level of paCO_2_ was considerable in the second ABGA. The increased level of paCO_2_ may reflect not only decreased alveolar ventilation, but also decreased lung perfusion and cardiac output^23^. Theoretically, the ETCO_2_ levels approximate paCO_2_ levels with normal pulmonary blood flow and ventilation. Conversely, during cardiac arrest, an abrupt decrease in cardiac output results in CO_2_ accumulation in the tissues while ETCO_2_ values decrease almost to zero^24^. ETCO_2_ has been investigated as a tool for monitoring CPR quality^25^, and also as a predictor of ROSC at the earlier stages of resuscitation; however, evidence shows that its accuracy is generally lower^26,27^. That might be the reason why the level of ETCO_2_ at 10 min of ED arrival did not reach statistical significance in terms of prediction of ROSC in our study. In light of the role as an early predictor of ROSC, the dynamic changes in paO_2_ between 0 and 10 min could provide additional information with the level of ETCO_2_. Therefore, this could be another piece of evidence to support the current guidelines on performing arterial catheterization during CPR^28–30^.

We selected the time of the second ABGA as 10 min after the start of CPR at the ED. An optimal time interval may exist and more frequent ABGAs may bring more significant results. However, given the need for a large amount of ED resources during CPR, it was reasonable to conduct ABGAs for the selected time interval in real clinical practice. Additionally, the third or fourth ABGA was performed in a few numbers of the patients, however, statistical analysis could not be conducted due to the small sample size.

The strength of our study is that all blood samples for gas analysis were obtained from confirmed arterial lines, and the confirmation was carried out in two ways: ultrasonography and arterial wave monitoring. However, most relevant studies have reported results of blood gas analysis, and not “arterial” blood gas analysis to date. Current CPR guidelines suggest that arterial blood pressure monitoring may guide physicians in assessing the quality of CPR to maintain the diastolic pressure above 20 mmHg^18,29,31^. Therefore, we attempt to place the arterial line during CPR as soon as OHCA patients arrive in our emergency department (ED). Serial measurement of arterial blood samples is possible after arterial catheterization.

We described the dynamic changes in arterial blood gas during CPR in a cohort of patients with OHCA; however, there were notable limitations to our study. First, there might be a possible bias in the selection of study patients. Only 80 patients out of 348 patients with non-traumatic OHCA were included in the present study and this was mainly due to the technical difficulties of arterial catheterization. Missing values of some parameters or lack of data of the second ABGA also contributed to exclusion. However, the rate of sustained ROSC in OHCA had been reported to be approximately 30% in previous studies, while only 16% of the patients achieved ROSC in our study^32^. Since patients whose second arterial blood samples were not obtained were excluded, those patients who achieved ROSC within 10 min of ED arrival were also naturally excluded from the study. In general, the resuscitation duration has been strongly associated with the outcomes of OHCA^33–35^; thus, interpretation of the results can vary if patients with relatively short durations of resuscitation are excluded from the study and if only patients with prolonged resuscitation are included therein. It was therefore not surprising that 16% of survival for a population with very prolonged resuscitation was attempted in our study. In Supplementary Table S1, the baseline characteristics and outcomes of patients are compared with respect to the placement of the arterial line. Actually, the median duration of total resuscitation was longer in patients with arterial line than in the other group of patients (46 vs. 40 min, *p* = 0.004). This could have contributed to the selection bias, and it could have resulted in a lower survival rate. Several known arrest characteristics, such as witnessed, bystander CPR, and presumed cardiac cause, might not achieve statistical significance due to these exclusions or small sample sizes^36^. Although the involved physicians were not blinded to the results from ABGA, they continued their resuscitation efforts for at least 20 min regardless of the results and provided standard treatments that complied with the guideline. Thus we believe that knowledge of the results of ABGA contributed very little to the selection bias. Second, our data showed that the blood gas analyses were performed mostly in the latter half of the resuscitation phase. The median prehospital downtime of the study population was 29 min, which was more than half of the median time of total resuscitation (51 min). This trend was also found in the groups when they were divided based on the achievement of sustained ROSC. Although there was no significant difference in the prehospital downtime between the sustained ROSC group and no-ROSC group, it is questionable whether our findings will be consistent in the early phase of cardiac arrests. Third, successful arterial catheterization itself could reflect a good prognosis. The chaotic situations in EDs may prevent a physician from placing an arterial line immediately. Even with an ultrasonography guide, a palpable pulsation can help in determining an appropriate catheterization site; otherwise, a palpable pulsatile flow in a peripheral artery could indicate that sufficient circulation was generated by chest compressions. Therefore, patients with an arterial line during chest compression may inevitably represent a positive selection. Owing to these limitations, only 80 patients were included in the study, which may have led to inadequately powered statistical analyses regarding the studied outcome. To overcome these limitations, acquisition of pre-hospital arterial blood samples and inclusion of patients from multiple centers should be considered accordingly.

## Conclusion

Our findings suggested that there was a possible association between the delta value of paO_2_ in ABGA during CPR and an increased rate of sustained ROSC in the late phase of OHCA. Hence, ΔpaO_2_ may serve as an independent prognostic value for sustained ROSC in OHCA.

## Supplementary Information

Supplementary Information.

## Data Availability

The datasets used and analyzed during the current study are available from the corresponding author on reasonable request.
