# Supplementary material for: Dynamic changes in arterial blood gas during cardiopulmonary resuscitation in out-of-hospital cardiac arrest
Source: Sci Rep. 2021 Nov 30;11:23165. doi: 10.1038/s41598-021-02764-4 (PMC8632901; doi:10.1038/s41598-021-02764-4)
Supplement: Supplementary file 1 — Supplementary Information. [file 41598_2021_2764_MOESM1_ESM.docx]

**Dynamic changes in arterial blood gas during cardiopulmonary resuscitation in out-of-hospital cardiac arrest**

Seok-In Hong, MD^1^, June-Sung Kim, MD, PhD^1^, Youn-Jung Kim, MD, PhD^1^, Won Young Kim, MD, PhD^1^*

^1^Department of Emergency Medicine, University of Ulsan College of Medicine, Asan Medical Center, Seoul, Korea

* Correspondence to: Won Young Kim, Department of Emergency Medicine, University of Ulsan College of Medicine, Asan Medical Center, Seoul 05505, Korea: Tel: +82-2-3010-3350, Fax: +82-2-3010-3360, Email: [wonpia73@naver.com](mailto:wonpia73@naver.com)

Supplementary Table S1. Baseline characteristics and outcomes of patients with and without placement of the arterial line

| **Characteristics**^a^ | **Patients without arterial line (n=131)** | **Patients with arterial line (n=217)** | ***p*-value** |
| --- | --- | --- | --- |
| **Arrest characteristics** | | | |
| Age (years) | 69 (56–80) | 72 (62–80) | 0.205 |
| Male sex | 93 (71) | 136 (63) | 0.151 |
| Witnessed | 86 (66) | 169 (78) | 0.015 |
| Bystander CPR | 69 (53) | 134 (62) | 0.133 |
| Prehospital use of mechanical compression device^b^ | 37 (28) | 54 (25) | 0.607 |
| In-hospital use of mechanical compression device^c^ | 54 (41) | 104 (48) | 0.255 |
| Prehospital shockable rhythm | 13 (10) | 26 (12) | 0.594 |
| Presumed cardiac cause | 40 (40) | 69 (32) | 0.237 |
| Prehospital downtime (mins)^d^ | 27 (22–34) | 28 (22–33) | 0.560 |
| Total resuscitation duration (mins) | 40 (32–50) | 46 (36–58) | 0.004 |
| ETCO_2_ (mmHg)^e^ | 12 (8–20) | 12 (9–20) | 0.541 |
| **Outcomes** |  |  |  |
| Any ROSC | 56 (43)) | 86 (40) | 0.646 |
| Sustained ROSC | 45 (34) | 56 (26) | 0.136 |

CPR, cardiopulmonary resuscitation; ETCO_2_, end-tidal carbon dioxide; ROSC, return of spontaneous circulation

^a^ Continuous variables are expressed as median with interquartile ranges; categorical variables are expressed as numbers with percentages.

^b^ LUCAS®, Lund University Cardiopulmonary Assist System; Physio-Control Inc./Jolife AB, Lund, Sweden or AutoPulse®; Zoll Medical Corporation, Chelmsford, MA, USA

^c^ LUCAS®

^d^ Defined as the estimated time from the first recognition of a sign of cardiac arrest to arrive in the hospital. The downtime in unwitnessed cases would be longer than the estimation.

^e^ The level of ETCO_2_ was measured at 10 minutes after ED arrival of the patient.
